# [Corrigendum] The mechanism of adenosine-mediated activation of lncRNA MEG3 and its antitumor effects in human hepatoma cells

**DOI:** 10.3892/ijo.2025.5765

**Published:** 2025-06-18

**Authors:** Li-Xuan Liu, Wei Deng, Xiao-Tao Zhou, Rui-Pei Chen, Meng-Qi Xiang, Yi-Tian Guo, Ze-Jin Pu, Rui Li, Ge-Fei Wang, Ling-Fei Wu

Int J Oncol 48: 421-429, 2016; DOI: 10.3892/ijo.2015.3248

Following the publication of the above article, an interested reader drew to the authors' attention that, for the fluorescence microscopic images shown in Fig. 4A on p. 425, the data shown for the 'Huh7 / pcDNA3.1-MEG3' experiment appeared to repeat some of the data shown in the 'HepG2 / pcDNA3.1-MEG3' experiment, albeit at a different magnification. Upon examining their original data, the authors realized that they had inadvertently assembled the data in this figure incorrectly.

The revised version of Fig. 4, now showing the correct images for the Control and pcDNA3.1-MEG3 experiments for the Huh7 cell line, is shown on the next page. The authors wish to state that these errors did not affect the overall conclusions reported in the study. The authors are grateful to the Editor of *International Journal of Oncology* for allowing them this opportunity to publish a Corrigendum, and all the authors agree with its publication. Furthermore, the authors apologize to the readership for any inconvenience caused.

## Figures and Tables

**Figure 4:**
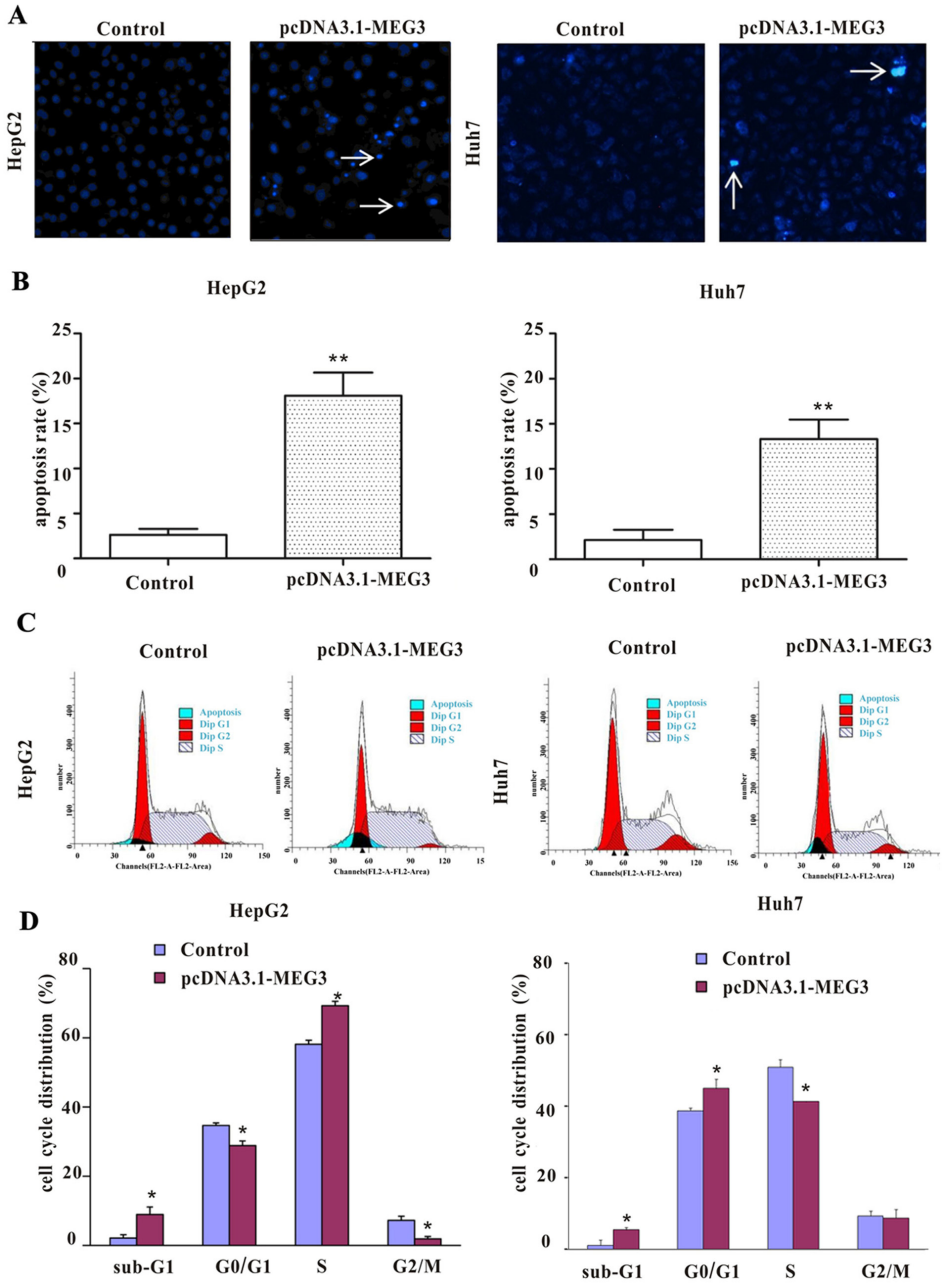
Effects of ectopic expression of MEG3 on apoptosis and cell cycles in the hepatoma cells. (A) The morphology of apoptotic nuclei was observed by Hoechst 33258 staining and fluorescence microscopy. The percentage of apoptotic nuclei per optical field was scored. (B) Quantitative analysis of the total apoptotic cells by fluorescence microscopy. Each bar corresponds to the mean ± SD of three independent experiments. (C) Cell cycles and apoptosis percentage were analyzed by flow cytometry and the apoptosis cells in sub-G1 phase (blue peak) were determined. (D) Quantitative analysis of the cell cycles. Bar chart represents the percentage of cells in G0/G1, S, sub-G1 or G2/M phase. ^*^p<0.05 vs. control; ^**^p<0.01 vs. control.

